# CD47 expression in solid tumors correlates with phagocytic tumor-associated macrophage gene signature

**DOI:** 10.3389/fimmu.2025.1699237

**Published:** 2025-12-03

**Authors:** Nicholas van Buuren, Mengshu Xu, Yi Zhang, Paola Correa, Shiva Zaboli, Azadeh Madjidi, Christina Moon, Szu-Wen Liu, Ruidong Li, Kai-Hui Sun, Shahed Iqbal, Abhishek Aggarwal, Min Wang, Li Li, Jared M. Odegard, Kelli Boyd

**Affiliations:** 1 Gilead Sciences Inc, Biomarker Sciences and Diagnostics, Foster City, CA, United States; 2 Immunome Inc, Biomarkers and Diagnostics, Bothell, WA, United States; 3 Gilead Sciences Inc, Research Data Sciences, Foster City, CA, United States; 4 Adept Computational Biology, Boerne, TX, United States; 5 Gilead Sciences Inc, Pathobiology Department, Foster City, CA, United States; 6 Routestone Consulting LLC, Seattle, WA, United States

**Keywords:** macrophage, Immune checkpoint inhibitor, solid tumor, phagocytosis inhibitor, tumor microenvironment - TME

## Abstract

**Background:**

CD47 is a “don’t eat me” signal that is overexpressed in tumors to evade phagocytosis by tumor associated macrophages (TAM). Investigational agents targeting CD47, such as magrolimab, aim to induce phagocytosis of tumor cells by TAMs. Previously, two key TAM subsets have been identified: C1QC TAMs, which display pro-phagocytic activity, and SPP1 TAMs that express pro-angiogenic markers. We characterize CD47 expression and its relationship with tumor macrophages in solid tumor samples.

**Patients and methods:**

Resectable tumors from head and neck squamous cell carcinoma (n=36) (HNSCC), breast cancer (n=37) (BC), and colorectal cancer (n=36) (CRC) were evaluated for CD47 expression by immunohistochemistry (IHC), two multiplex immunofluorescence panels were used to characterize TAM markers and T cell markers. RNA sequencing was also performed.

**Results:**

CD47 protein expression was higher on tumor cells compared to stromal cells across tumor indications tested. Although CRC had the lowest prevalence for CD47 expression in primary tumors, we observed a marked increase in CD47 expression in CRC liver metastases. We developed an SPP1 TAM gene signature and validated a C1QC TAM gene signature to estimate TAM abundances from bulk RNA-Seq data. In the TAM mIF analysis, HNSCC had the highest macrophage density of the indications tested. We observed a positive correlation between a higher C1QC: SPP1 TAM ratio and macrophage phenotype and tumor T cell density. C1QC macrophage signatures correlate with tumor CD47 protein expression in BC and HNSCC samples, suggesting interplay between them.

**Conclusions:**

We characterized CD47 expression across key solid tumor indications being evaluated clinically using anti-CD47 blockade agents: HNSCC, breast cancer and CRC. Using a CD47 IHC assay, we identified HNSCC as an indication with the highest CD47 expression. In addition, we quantified tumor macrophages using multiplex immunofluorescence (mIF) and determined that HNSCC had the highest density of TAMs. Compared to relatively low CD47 expression in primary CRC tumors, CRC liver metastases had very high CD47 expression. Quantification of TAM signatures and CD47 expression represent key biomarkers to monitor in patient samples during exploration of CD47-blockade agents in the clinic.

## Introduction

CD47 is a “don’t eat me” signal expressed on tumor cells to prevent phagocytosis by tumor macrophages. CD47 antagonizing antibodies, such as magrolimab, have been shown to promote phagocytosis of tumor cells through disruption of the interaction between macrophage SIRPα and tumor CD47 (1–3). Investigation of CD47 blocking agents for the treatment of solid tumors has demonstrated mixed results across a variety of indications and further development continues to follow up on these early signals (4–6).

CD47 expression in solid tumor samples has been characterized using a variety of IHC assays and high expression is typically associated with poor prognosis (7–10). These data indicate that CD47 expression is increased on tumor cells and that CD47 expression is highly heterogeneous within indications. CD47 expression represents a key biomarker to monitor in response to anti-CD47 blockade. Here we developed a CD47 IHC assay with high specificity for CD47 and low levels of background staining to quantify the prevalence of CD47 expression in HNSCC, BC and CRC.

CD47 blockade is hypothesized to trigger tumor cell phagocytosis by TAMs. Historically, macrophages have been classified into pro-inflammatory M1 or immuno-suppressive M2 subtypes. Recently, scRNA-Seq studies on tumor samples have failed to identify any macrophage subsets that fit the M1/M2 paradigm. Instead, many macrophage subtypes express mixtures of M1 and M2 markers (11–13). scRNA-Seq studies have shown that tumor macrophage subsets are highly phenotypically plastic and show a higher level of diversity and cancer type specificity compared to other immune cell types such as lymphocytes. A diverse array of macrophage subtypes has been described across normal tissues and solid tumor indications. Here, we focus on C1QC TAMs, which are pro-phagocytic and SPP1 TAMs, which are pro-angiogenic (10, 13, 14). Both macrophage subtypes are enriched in tumors, although C1QC macrophages are also found in normal tissues (11). We validate a C1QC TAM gene signature suitable for use in bulk RNA-Seq in HNSCC, BC and CRC FFPE tumor samples. Additionally, we developed a macrophage-specific SPP1 TAM gene signature suitable for bulk RNA data.

Preclinical data indicate that the mechanism of action of CD47 blockade might involve downstream activation of T cells through macrophage-mediated antigen presentation following tumor cell phagocytosis (15, 16). To characterize both TAM infiltration and its relationship to the T cell compartment, we developed two mIF panels which were used to phenotype and localize TAMs and T cells within the TME.

## Materials and methods

### Sample collection

Solid tumor resections for primary HNSCC (n=44), BC (n=48), and CRC (n=48) were commercially procured from BioIVT and Precision for Medicine. These sampling numbers was chosen based on the central limit theorem (n >=30) with additional cases in case of sample attrition due to technical reasons. BC FFPE samples were originally procured as TNBC breast cancer specimens; however, due to their archival nature, many lacked definitive clinical annotations of receptor status (ER, PR, HER2). Therefore, we refer to them broadly as breast cancer (BC) throughout the manuscript. However, we further classified BC cases molecularly using the PAM50 transcriptional signatures as Basal-like, Luminal A, and Her2 positive (**Supplementary Figure 4A**). In addition, forty FOLFOX-experienced (combination folinic acid/leucovorin calcium, fluorouracil and oxaliplatin chemotherapy) primary CRC tumor samples and 48 FOLFOX-experienced CRC liver metastasis samples were procured from Cureline Group (Brisbane, California). Patients who received radiation within 1 year of surgical sample/biopsy collection, have mutations of known BRAF V600E, MSI-H, or dMMR genes, or had prior irinotecan therapy were excluded. The time between initiation of FOLFOX treatment to collection of tumor tissues is six months or less.

### Immunohistochemistry

Immunohistochemistry (IHC) for CD47 was conducted on a 5μm section from each sample on the DISCOVERY ULTRA instrument from Roche Tissue Diagnostics (Oro Valley, AZ).

Antigen retrieval was performed with CC1 solution for 32 minutes. Staining was done using rabbit anti-CD47 clone EPR21794 (Abcam, cat# ab218810, Rabbit IgG, 1.1 ug/ml final concentration) and Biocare Da Vinci Green Diluent as diluent for 60 minutes at room temperature. IHC slides were either scanned with a Leica Aperio AT2 (Leica Biosystems) prior to digital pathology analysis or scored manually by a pathologist.

IHC for SIRPα was performed on the Leica Bond RX autostainer (Leica Biosystems, Deer Valley, IL). Antigen retrieval was done with Leica Biosystems’ BOND Epitope Retrieval Solution 2 (ER2) at 95°C for 20 minutes. Antibody staining utilized rabbit anti-SIRPα (LifeSpan BioSciences, LS-B551, polyclonal, Rabbit IgG, 2.5ug/ml final concentration) and Leica Bond Diluent as diluent for 20 minutes at room temperature. IHC slides were scanned with a Leica Aperio AT2 (Leica Biosystems) prior to digital pathology analysis.

### HPV HR8 RNAscope assay

HNSCC samples were evaluated for human papilloma virus using High Risk HPV RNAscope™ ISH Probe (Advanced Cell Diagnositics, Newark, CA; Cat# 200450) which qualitatively detects E6/E7 mRNA of high-risk HPV in FFPE tissue specimens. Manufacturers protocol was followed for this assay.

### FFPE RNA-seq: RNA extraction, library construction, and sequencing

RNA was extracted from four 10µm sections from each FFPE block using the Qiagen AllPrep FFPE Kit (Cat no. 80234; Qiagen, Germantown, MD, USA) following the manufacturer’s protocol.

For RNA-Seq, RNA concentration and integrity were assessed using the Qubit fluorometer with the RNA High Sensitivity Assay Kit (Cat no. Q32852; ThermoFisher Scientific, Waltham, MA, USA) and using the Fragment Analyzer with Agilent High Sensitivity RNA Kit (Cat no. DNF-472; Agilent Technologies, Santa Clara, CA, USA). RNA was used as an input material for library construction using Illumina RNA Prep with Enrichment (L) Tagmentation Kit with the Illumina Exome Panel (Cat no. 20040537 and Cat no. 20020183; Illumina, San Diego, CA, USA) following the manufacturer’s protocol.

Library quality control was performed to assess both the concentration and size distribution of the libraries. The library concentration was determined using the Qubit fluorometer with the DNA High Sensitivity Assay Kit (Cat no. Q32851; ThermoFisher Scientific, Waltham, MA, USA), and the library size was confirmed using the 2200 TapeStation with the D1000 ScreenTape (Cat no. 5067-5582; Agilent Technologies, Agilent Technologies, Santa Clara, CA, USA). The libraries were multiplexed and then sequenced on either the Illumina NextSeq2000 or NovaSeq6000 (Illumina, San Diego, CA, USA), generating 2 x 100nt-paired end reads with a mean aligned pair-read count of 28 million reads per sample.

### Data processing: quality control and gene expression profiling

For RNA-Seq, a Nextflow (17) pipeline was developed for quality control and gene expression profiling. Multiple quality control steps were performed using FastQC (v0.11.8) (18), RNA-SeQC2 (v2.4.2) (19)and MultiQC (v1.10.1) (20) to ensure high quality sequencing data for downstream analysis. Sequencing reads were aligned to Genecode GRCh38.v38 reference genome using STAR (2.5.3a) (21). Gene-wise TPM and expected read counts were generated using RSEM (v1.3.1) (22). TPM expression signals were normalized between samples by quantile normalization using limma (23). Samples that passed RNA-seq quality control filtering included primary HNSCC (n=36), BC (n=37), and CRC (n=36). RNA-seq raw data is available at SRA accession PRJNA1261839 and processed data is available at NCBI GEO (GSE296861).

### Cancer molecular subtype classification

Primary CRC samples were classified into CMS classes by running CMScaller (24, 25) on the RNA-Seq data.

The ISH HPV assay was used to determine status in 33 HNSCC samples out of 36; however, two samples were removed from analysis due to technical reasons, and one sample failed QC. To provide a secondary HPV call for these samples, a published eight-gene HPV+ signature was used to predict the HPV status from the HNSCC RNA transcriptome data (26) (**Supplementary Figure 4B**). A comparison of the CISH-based HPV calls and HPV transcript signature score shows agreement in 4 out of 5 CISH HPV+ samples and 31/31 CISH HPV- samples. We therefore used the HPV signature score to infer the HPV status of the 3 samples which failed CISH HPV testing.

BC samples were hierarchically clustered based on PAM50 marker expression and we found that they were clearly separated into three clusters with expected subtype marker expression. Of 37 cases, 24 were Basal-like, 6 were HER2-enriched, and 7 were Luminal A (**Supplementary Figure 4A**). While most TNBC cases fall into the Basal-like molecular subtype, the overlap is imperfect. Luminal A and HER2-enriched molecular subtypes are mutually exclusive with TNBC by clinical definition (ER-/PR-/HER2-).

### Multiplex immunofluorescence

Two multiplex immunofluorescence (mIF) panels were developed using the tyramide signal amplification (TSA) based Opal platform (Akoya Biosciences) and performed on a Bond RX autostainer (Leica Biosystems). The macrophage panel was built as follows: Position 1: anti-PD-L1 (Abcam ab228415, clone 73-10, Rabbit IgG, 1ug/mL final concentration) with Opal 520 Fluor at 1:150 dilution; Position 2: anti-CD68 (Invitrogen MA1-80133, clone 514H12, Mouse IgG2a, 1:200 dilution) with Opal 570 Fluor at 1:150 dilution; Position 3: anti-CD163 (Abcam EPR19518, clone EPR19518, Rabbit IgG, 0.2ug/mL final concentration with Opal 620 fluor at 1:150 dilution; Position 4: anti-pan-Cytokeratin (Abcam ab215838, cocktail: no clone, Mouse IgG, 2ug/mL final concentration) and TSA-DIG/Opal 780 Fluor at 1:150/1:25, respectively.

The resident memory T cell panel consisted of: Position 1: anti-CD103 (Abcam ab224202, clone EPR22590-27, Rabbit IgG, 2ug/mL final concentration) with Opal 520 Fluor at 1:150 dilution; Position 2: anti-CD8 (Thermo Fisher MA1-80231, clone 4B11, Mouse IgG1k, 1:100 dilution) with Opal 570 Fluor at 1:150 dilution; Position 3: anti-CD3 (Abcam ab16669, clone SP7, Rabbit IgG, 1:100 dilution) with Opal 620 Fluor at 1:150 dilution; and the same anti-pan-Cytokeratin described above at Position 4. Spectral DAPI (PerkinElmer FP1490, 2 drops/mL in TBS buffer) was used to counterstain. Slides were scanned to generate whole slide images (WSI) using the PhenoImager HT (Akoya Biosciences).

### Image analysis

CD47 IHC digital images were quantified using Visiopharm software (version 2023.01, Visiopharm, Denmark). To measure CD47 expression in both tumor and non-tumor regions, we utilized two serial sections of the same sample, stained for CD47 and cytokeratin (CK). A threshold-based approach was used to identify areas with a positive CD47 signal, enabling us to calculate the percentage of CD47 expression in both compartments. Expression was classified into four categories: negative, low, medium, and high and used to calculate the H-score for both tumor and non-tumor regions. In parallel, digital images were assessed manually by a pathologist at Mosaic Laboratories (CellCarta: Lake Forest, CA) for % of neoplastic cells with CD47 membrane staining and a corresponding H-score.

Whole slide mIF images were analyzed using Visiopharm software (version 2023.01, Visiopharm, Denmark). A threshold-based algorithm was implemented to detect the tissue from the background glass slide by aggregating the signal across all existing channels in the panel at 1x magnification. Another threshold-based algorithm was applied to the panCK channel to detect the tumor regions. Next, a convolutional neural network (CNN; U-Net) was trained and applied at 20× magnification to segment and classify nuclei using the DAPI channel in both compartments (tumor or non-tumor). Finally, using a threshold-based approach, cells were classified as positive or negative for each marker, and phenotypes derived.

### Real world evidence analysis using TempusAI

Real-world data were obtained from the TempusAI multimodal database, which integrates clinical and multi-omic information from a variety of healthcare environments. Transcriptomic profiling was conducted using the Tempus xR next-generation sequencing platform. As of December 2023, the Tempus database contained approximately 7.7 million clinical records, of which 910,000 included both molecular (DNA and/or RNA) and clinical data. From this subset, we licensed access to de-identified patient cohorts with metastatic disease, defined as stage IV or M1 according to the AJCC 8th edition criteria, although many of the RNA sequencing samples come from primary tumor sites. The selected cohorts comprised patients with breast cancer (n = 55), head and neck cancer (n = 266), and microsatellite stable (MSS) colorectal cancer (n = 1,051). All patients had undergone bulk RNA sequencing and had documented clinical outcomes following first-line therapy. Patients that were sampled prior to later line treatments were omitted from the analysis to ensure our prognostic evaluation was not influenced by stage of disease.

## Results

### CD47 expression is upregulated in solid tumors

**Figure 1:**
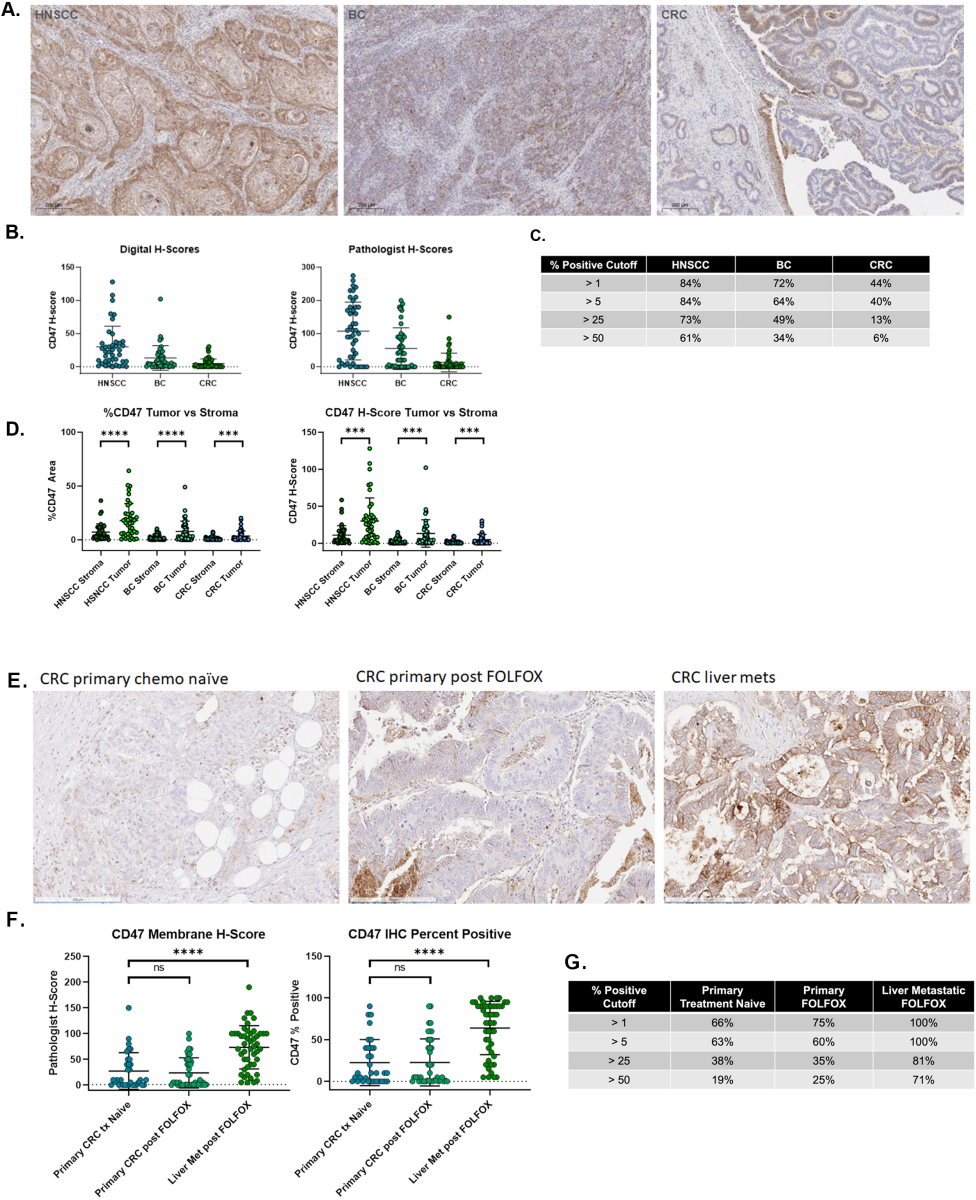
CD47 is upregulated on tumor cells in HNSCC, BC and CRC. **(A)** Slides were stained with anti-CD47 (clone EPR21794) for immunohistochemistry analysis. **(B)** CD47 expression was scored using a digital algorithm as well as manual pathologist scoring on tumor cell membranes. **(C)** Prevalence for CD47 expression was determined at a variety of cutoffs using % cells positive for CD47 expression by manual pathologist scoring algorithm. **(D)** CD47 expression in tumor versus stroma was calculated using the digital algorithm and plotted as H-score and % positive area. **(E)** Slides were stained with anti-CD47 (clone EPR21794). **(F)** Manual pathologist assessment of CD47 expression on tumor cell membranes reported as H-score and % cell positive. **(G)** Prevalence for CD47 expression was determined at a variety of cutoffs using % cells positive for CD47 expression by manual pathologist scoring algorithm. Statistical analyses performed using t-test. P values: * p < 0.05, ** p < 0.01, *** p < 0.001 and **** p < 0.0001.

FFPE samples of primary, resectable tumors from patients with HNSCC (N = 44), BC (N = 48) and CRC (N = 48) were evaluated for CD47 expression by IHC (**Figure 1A**). Images were analyzed by both a digital algorithm and by manual pathologist derived annotations to score CD47 expression (**Figure 1B**). These scoring metrics were highly concordant (paired Spearman rho=0.77, p value = 2.2x10^-16^), although the pathologist derived H-scores showed a larger dynamic range **(Supplementary Figure 1**). All future staining for CD47 expression was scored only by the pathologists H-score method as this did not require a serial cyto-keratin-stained slide and would be easier to score on clinical samples when they became available. CD47 was heterogeneously expressed throughout all three solid tumor indications as previously reported (7, 8). Prevalence was assessed using the percentage of tumor cells that were positive for CD47 membrane scoring above a variety of cutoffs. Using >1% as the cutoff, HNSCC (84%) has the highest prevalence for CD47 expression, followed by BC (72%) and CRC (44%) (**Figure 1C**). CD47 expression measured with the digital algorithm demonstrated increased expression on tumor cells compared to the surrounding stromal cells (**Figure 1D**).

While CRC primary tumors had the lowest relative CD47 expression, we sought to determine if expression may be increased by treatment or by liver metastasis. The liver is the most common site of distant spread for CRC; up to 50% of patients have liver metastases at the time of primary diagnosis or develop them within 5 years (27, 28). We used the CD47 IHC assay to quantify CD47 protein expression in liver metastatic CRC samples and scored them by manual pathologist H-score (**Figure 1E, F**). CD47 IHC signal was comparable between primary treatment-naïve and FOLFOX-treated patients, implying FOLFOX *per se* does not affect CD47 expression in CRC tumors, however, significantly higher CD47 staining was detected in liver metastases (**Figure 1F**). Increased staining comprised both intensity and prevalence, which was 100% in the liver metastases (as defined as >1% CD47 positive tumor cells) (**Figure 1G**).

Next, we utilized the Tempus real world data to probe the prognostic value of CD47 gene expression (**Supplementary Figure 3A**). Notably, in HNSCC and BC, patients with higher than median CD47 gene expression demonstrated a trend towards poorer survival that was not significant. No such trend was observed in CRC. Given the high levels of CD47 expression in CRC liver metastasis compared to primary tumors, we utilized the Tempus RWE dataset to reinvestigate the prognostic impact of CD47 once tumor location was accounted for. However, we did not observe a statistically significant prognostic effect when assessing PFS after separating primary and liver metastasis samples (**Supplementary Figure 3B**).

### Macrophage infiltration is highest in HNSCC tumors

**Figure 2:**
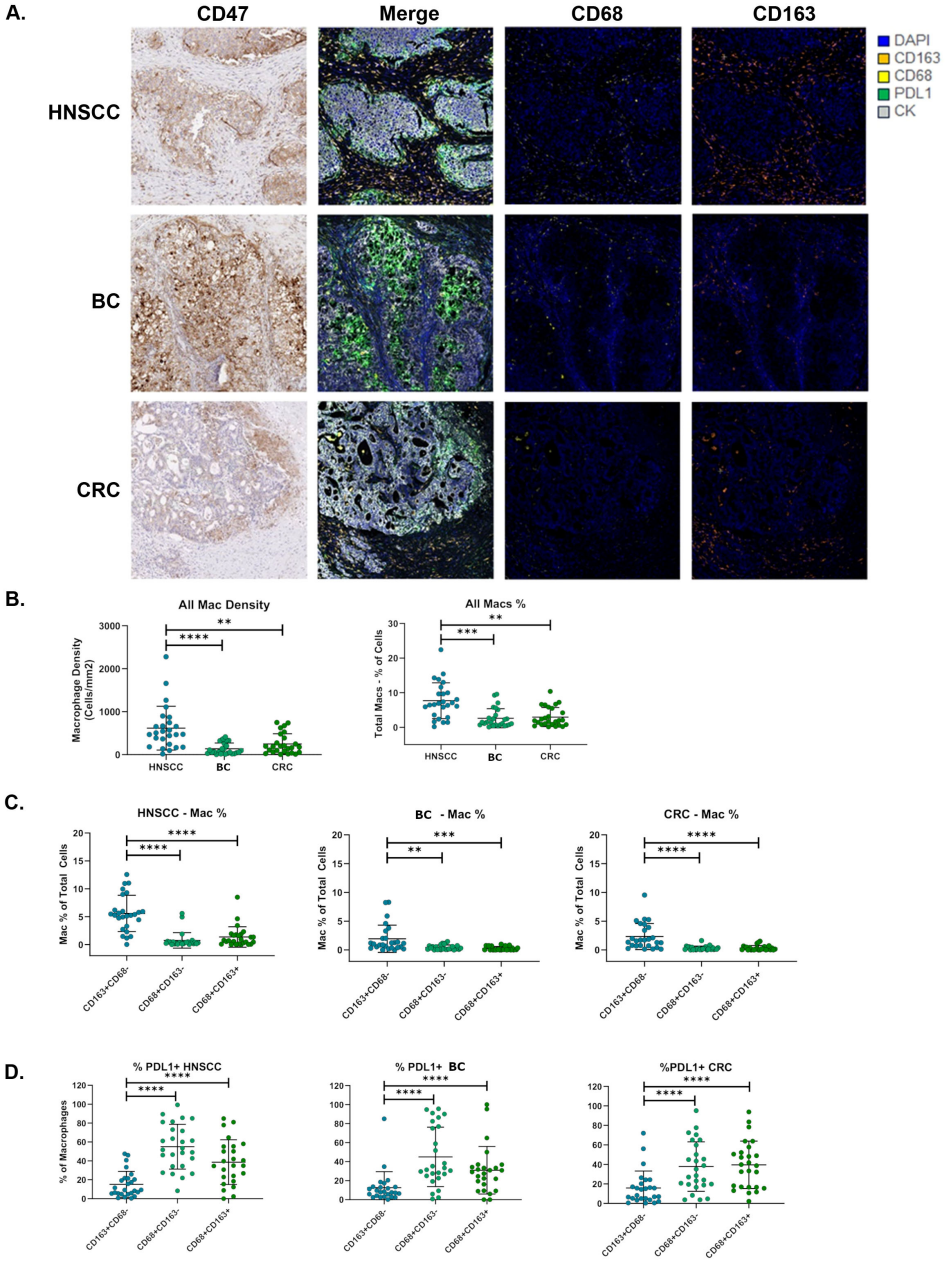
CD163+/CD68- macrophages are the most abundant TAM subgroup and express low levels of PDL1. **(A)** Tumor macrophages were stained with a 4-plex mIF panel consisting of CD68, CD163, PD-L1 and cytokeratin for multiplex immunofluorescence analysis. **(B)** TAMs were quantified based on co-expression patterns of all markers to quantify TAM phenotypes. Total TAMs were quantified by calculating the number of cells positive of either CD68 or CD163 and normalized by tissue area (left) or total cell number (right). **(C)** Individual TAM subsets, CD163+/CD68-, CD163-/CD68+ and CD163+/CD68+ were quantified as a percentage of total cells on each slide. **(D)** PD-L1 positive TAMs were quantified and normalized by percentage of each TAM phenotype. Statistical analyses performed by unpaired t-test in **(B)** and by paired t-test in **(C, D)** P values: * p < 0.05, ** p < 0.01, *** p < 0.001 and **** p < 0.0001.

To evaluate TAM abundance across HNSCC, BC and CRC tumor samples, we developed a 4-plex mIF assay to detect CD68, CD163, PD-L1 and cytokeratin (**Figure 2A**). As SIRPα is a key ligand for CD47, we also attempted to develop a SIRPα IHC assay that could be incorporated into this mIF panel. However, our SIRPα antibody demonstrated broad expression across tumor cells, lymphocytes, and macrophages. Thus, we discontinued assay development due to its highly non-specific staining (**Supplementary Figure 2**). Using the 4-plex mIF assay, we characterized TAMs across all three indications by quantifying cells positive for either CD68 or CD163 (**Figure 2B**). HNSCC was identified as the indication with the highest level of TAMs in the tumor microenvironment, which is consistent with HNSCC being widely known as an immune-rich indication. CD68 is widely used as a pan-macrophage marker and CD163 as a “M2-like” macrophage subset marker; however, we observed that CD68^+^ and CD163^+^ TAM subsets were largely distinct. CD163^+^CD68^-^ cells were the most abundant TAM population across all three indications (**Figure 2C**). These cells were slightly more prevalent in the stroma compared to the tumor, while TAMs were not excluded from the tumor nest (data not shown). When evaluating PD-L1 expression on the TAM subsets, we observed that CD163^+^CD68^-^ TAMs had the lowest prevalence of PD-L1 staining, while any CD68^+^ TAMs (CD68^+^/CD163^+^ or CD68^+^/CD163^-^) demonstrated roughly 2-fold higher prevalence for PD-L1 expression compared to those that are CD163^+^/CD68^-^ (**Figure 2D**).

### SPP1 TAM and C1QC TAM gene signatures for characterization of TAMs in bulk mRNA-Seq analysis

To further characterize phenotypes of TAMs in our tumor samples, we referenced previously described TAM subtypes in literature, particularly in recent scRNA-Seq studies (11, 13). C1QC TAMs express phagocytosis functional markers and SPP1 TAMs express angiogenesis markers. These TAM subtypes are named for a prominent expression marker and not for their inferred functions as to acknowledge potential functions that are yet unknown. The relative abundance of these two TAM populations can predict patient outcomes in colorectal and cervical cancers (11, 13). Based on markers validated in CRC from a Zhang et al. study (11), we looked for C1QC and SPP1 TAM signatures that could be used to estimate the abundance of the respective TAM populations in bulk sequencing data. While the Zhang et al. study used SPP1 TAM and C1QC signatures in their analysis, they did not explicitly name sets of markers that constituted a C1QC TAM and a SPP1 TAM signature; therefore, we selected markers that were prominently and repeated mentioned in the paper and validated their utility as signature markers. We found that while the C1QC TAM signature markers (APOE, C1QC, C1QB, C1QA, TREM2, MRC1, CD163, MERTK) contain genes that are specific for macrophages, some markers attributed to SPP1 TAMs (SPP1, PPARG, ADM, MARCO, VEGFA, VCAN, CXCL8, ANGPTL4) are also highly expressed in tumor and stromal cells, making them inappropriate to detect SPP1 TAMs from bulk sequencing data (**Supplementary Figure 5A**). We therefore developed a new macrophage-specific SPP1 TAM signature (PLAUR, SLC11A1, BRI3, FBP1, C15orf48) using publicly available scRNA-Seq data from lung, colorectal and breast cancers (14) and validated the signature using multiple independently published scRNA-Seq datasets (**Supplementary Figure 5B**). Briefly, scRNA data from Qian et al. contained author annotated immune and stromal cell types, including SPP1 TAMs and C1QC TAMs. We identified highly expressed SPP1 TAM markers by differential expression analysis comparing SPP1 TAMs against all other cell types. We further filtered for markers that are modestly expressed in other macrophage cell types and minimally expressed in all other cell types. This involved checking marker expression in scRNA-Seq reference datasets from the Human Protein Atlas and Tabula Sapiens (29–31). Candidate markers were cross-validated on two scRNA-seq datasets: clear cell renal carcinoma data and colorectal carcinoma data (11, 32). To test whether signature can detect relative levels of SPP1 TAM in bulk RNA-Seq data, we generated pseudo-bulk RNA samples from scRNA-Seq data into which we added various low percentages (0 to 2.5%) of either general macrophages, C1QC TAMs, or SPP1 TAMs with a fixed mixture of non-macrophage cell types. Low TAM percentages were used to model challenging bulk RNA-Seq samples with high tumor cell content and low immune cell abundance. We calculated the SPP1 TAM signature score for each pseudo-bulk sample by computing the mean z-score of signature genes. As a positive control, we also tested the original SPP1 TAM signature from Zhang el. al. (**Supplementary Figure Supplementary 5B** shows a representative test on Zhang Smart-Seq2 data (11)). Both signatures were most sensitive to presence of SPP1 TAMs, but also detected other macrophages to a lesser extent. This validation data shows that the new SPP1 TAM signature is at least as sensitive and specific to SPP1 TAM cells as previous versions; however, it is more suitable for use with bulk sequencing samples that contain tumor and stromal cells since it does not pick up signal from those other cell types. In addition, we used the same pseudo-bulk data to validate the specificity of the Zhang el al. C1QC TAM signature and find that it is very specific and sensitive for C1QC TAMs (**Supplementary Figure Supplementary 5B** right).

**Figure 3:**
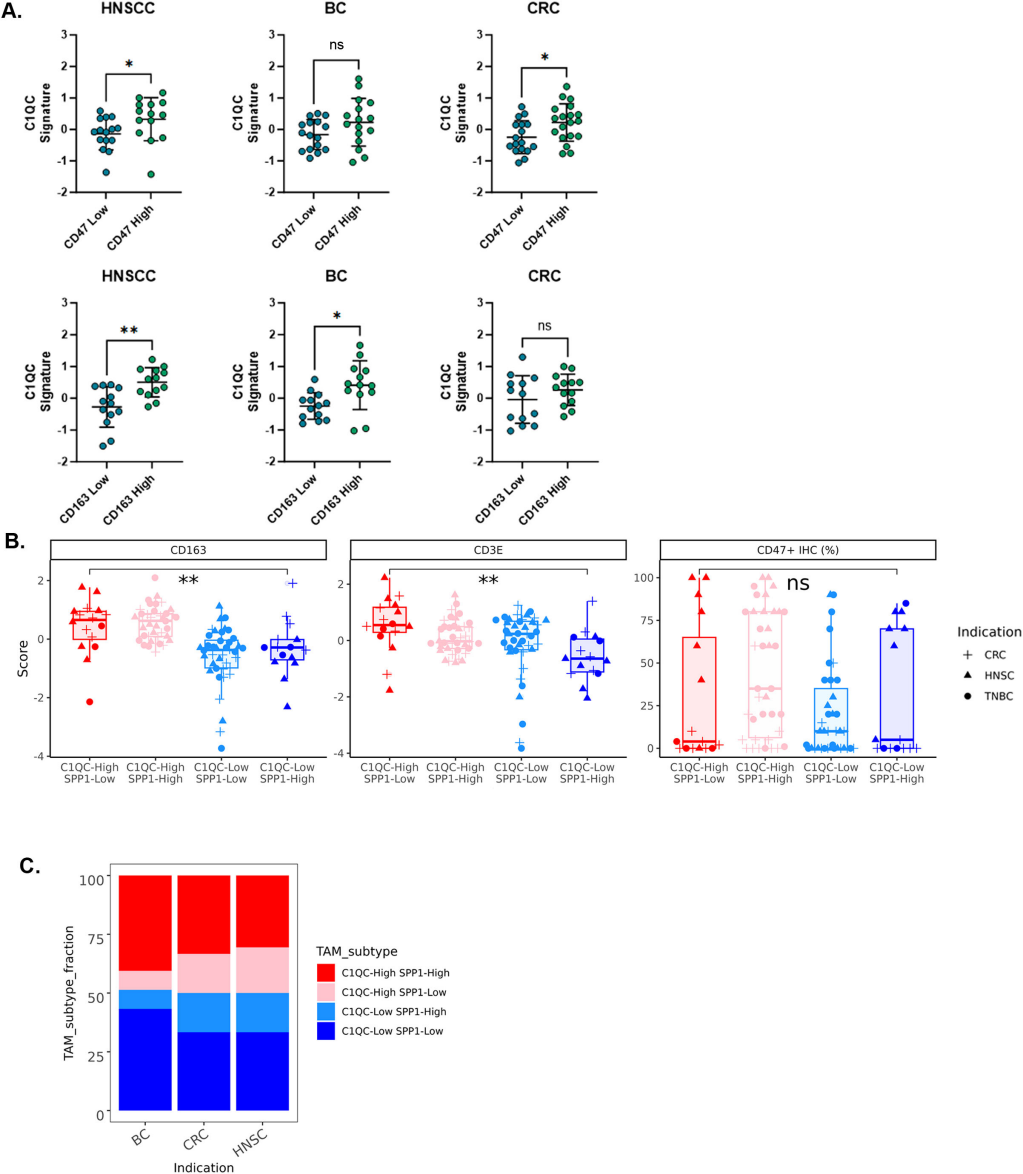
High CD47 expression is associated with a higher C1QC gene signature score. **(A)** Samples from individual indications binned as CD47-high or CD47-low at the median % positive, or CD163-high or CD163-low at the median CD163 TAM density and the C1QC gene signature score was plotted. **(B)** Four TAM bins consisting of samples from HNSCC, BC and CRC were created using C1QC and SPP1 gene signature scores. CD47 IHC, CD163 mRNA and CD3E gene expression were quantified for each TAM bin. **(C)** The percentage of samples that fell into individual TAM bins was plotted for HNSCC, BC and CRC sample types. Statistical analyses performed using t test. P values: * p < 0.05, ** p < 0.01, *** p < 0.001 and **** p < 0.0001.

To test the relationship between tumor macrophages and CD47 expression, we evaluated the correlation between the TAM gene signatures and CD47 IHC data on samples that passed RNA-Seq quality control (HNSCC (n=36), BC (n=37), and CRC (n=36)). Samples were stratified at the median CD47 expression level (by percent tumor cells positive) into CD47-high and CD47-low bins, and the C1QC gene signature score was plotted for each sample across indications (**Figure 3A**). The C1QC signature score demonstrated a statistically significant increase in CD47-high samples in HNSCC and CRC, and trended higher in BC (**Figure 3A**). No correlation between the SPP1 gene signature and CD47 expression was observed (data not shown). In addition, we stratified samples using the CD163 marker from our mIF panel at the median cell density. We observed that the C1QC TAM signature score was statistically higher in samples with high CD163 cell density in HNSCC and BC, and trended higher in CRC (**Figure 3A** bottom). No correlation was observed between CD163 positive macrophages and the SPP1 gene signature.

As the ratio between C1QC and SPP1 signatures has been shown to be associated with lymphocyte infiltration and prognosis in CRC (11), we tested whether CD47 (IHC), CD163 (gene expression) or CD3 (gene expression) correlated with the C1QC: SPP1 TAM ratio. Samples were divided at the median for SPP1 and C1QC signature scores to create four bins: C1QC-high:SPP1-low, C1QC-high:SPP1-high, C1QC-low:SPP1-high and C1QC-low:SPP1-low. Consistent with previous reports, samples that were C1QC-high:SPP1-low had the highest T cell content as quantified by CD3E gene expression (**Figure 3B**, and by CD8A expression **Supplementary Figure 5C**). We also observed that C1QC-high samples, independent of SPP1 status, demonstrated higher CD163 mRNA expression. Finally, no correlation was observed between the ratio of C1QC: SPP1 and CD47 expression, indicating that C1QC TAMs are driving the correlation with CD47 expression independent of SPP1 abundance. Finally, we assessed the prevalence of the four TAM subtype bins within each indication and did not observe differences between the three indications (**Figure 3C**).

### T cell infiltrate across solid tumors

**Figure 4:**
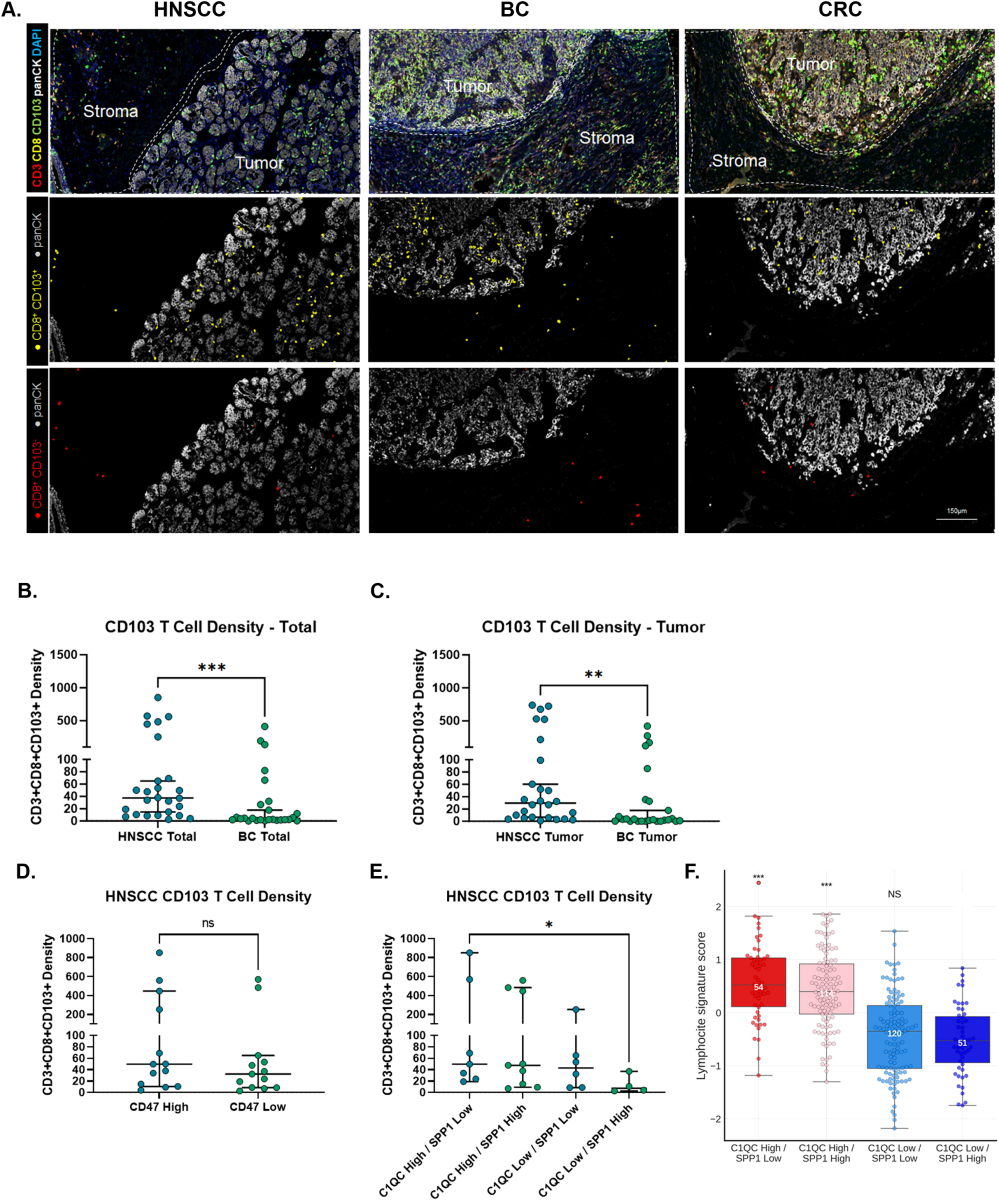
CD103+ T cells are highest in tumor samples with high C1QC and low SPP1 TAM populations. **(A)** A four-plex mIF panel was constructed to quantify CD3, CD8, CD103 and cytokeratin in solid tumor samples. Depicted are representative images demonstrating CD3+CD103+ T cell density (yellow) and CD3+CD103- T cell density (red) across HNSCC, BC and CRC samples. **B/C.** Density of CD3+CD103+ T cells in each sample, or specifically in the tumor, was calculated using total area **(B)** and tumor area **(C)** as the denominator. **(D)** CD47 expression in HNSCC samples was stratified at the median using percent positive and CD3+CD103+ T cell tumor density was plotted for CD47-high and CD47-low samples. **(E)** TAM classifications determined using RNA-Seq data were assigned to each HNSCC sample. CD3+CD103+ T cell density in the tumor was plotted for each of the four TAM bin classifications. **(F)** Tempus RWE was used to evaluate a CD8 T cell gene signature across the four TAM bins. Groups were compared to C1QC low/SPP1 high bin. Statistical comparisons by Mann-Whitney U test; p-values: NS p>=0.05, *p<0.05, **p<0.005, ***p<0.005.

To further evaluate the relationship between TAMs and tumor specific T cells, we developed a 4-plex mIF assay consisting of CD3, CD8, CD103 and cytokeratin (**Figure 4**). CD103 is a marker of tissue residency, and together with CD8, they mark resident memory CD8 T cells (Trm) which are typically tumor antigen specific (33–35). The majority of CD103^+^ T cells (yellow) were found inside the tumor, while CD103^-^ T cells (red) were found more commonly in the surrounding stroma (**Figure 4A**). The density of CD103^+^ T cells was highest in the tumor nest of HNSCC samples compared to BC samples in our dataset (**Figure 4, C**).

Next, we evaluated the relationship between the density of CD103^+^ T cells and CD47 (protein IHC) expression as well as the TAM ratio in the HNSCC and BC sample sets. In both HNSCC (**Figure 4D**) and BC (data not shown) there was no statistically significant correlation between CD103^+^ T cell density and CD47 expression. However, we observed a correlation between the TAM gene signature ratios and CD103^+^ T cell density in HNSCC (**Figure 4E**). We further evaluated this correlation in a large cohort of HNSCC samples from the Tempus RWE dataset and found the same relationship between the TAM gene signature ratio and a CD8 lymphocyte signature score (CD8A, CD8B, GZMB) (**Figure 4F**). A similar positive relationship between C1QC TAMs and T cell infiltration has been previously described in CRC and is thought to be associated with the mechanism for the prognostic impact of the C1QC TAM gene signature (11).

### Tumor molecular subgroups have modest differences in CD47 expression and TAM compartments

To evaluate CD47 expression and TAM biology within various tumor molecular subtypes, we used the tumor sample transcriptome to stratify samples into known molecular subtypes for CRC, HNSCC and BC (**Supplementary Figure 4A**). For CRC, these molecular subgroups consist of CMS1, CMS2, CMS3 and CMS4 which have been described previously (24, 36, 37). BC samples were grouped into Luminal A, Her2+ and Basal-like subtypes using PAM50 classification (no Luminal B samples were found in our dataset) (38–40). We divided the HNSCC samples based on HPV infection status using both a HPV ISH assay and HPV status transcriptional signature (**Figure 4B**).

**Figure 5:**
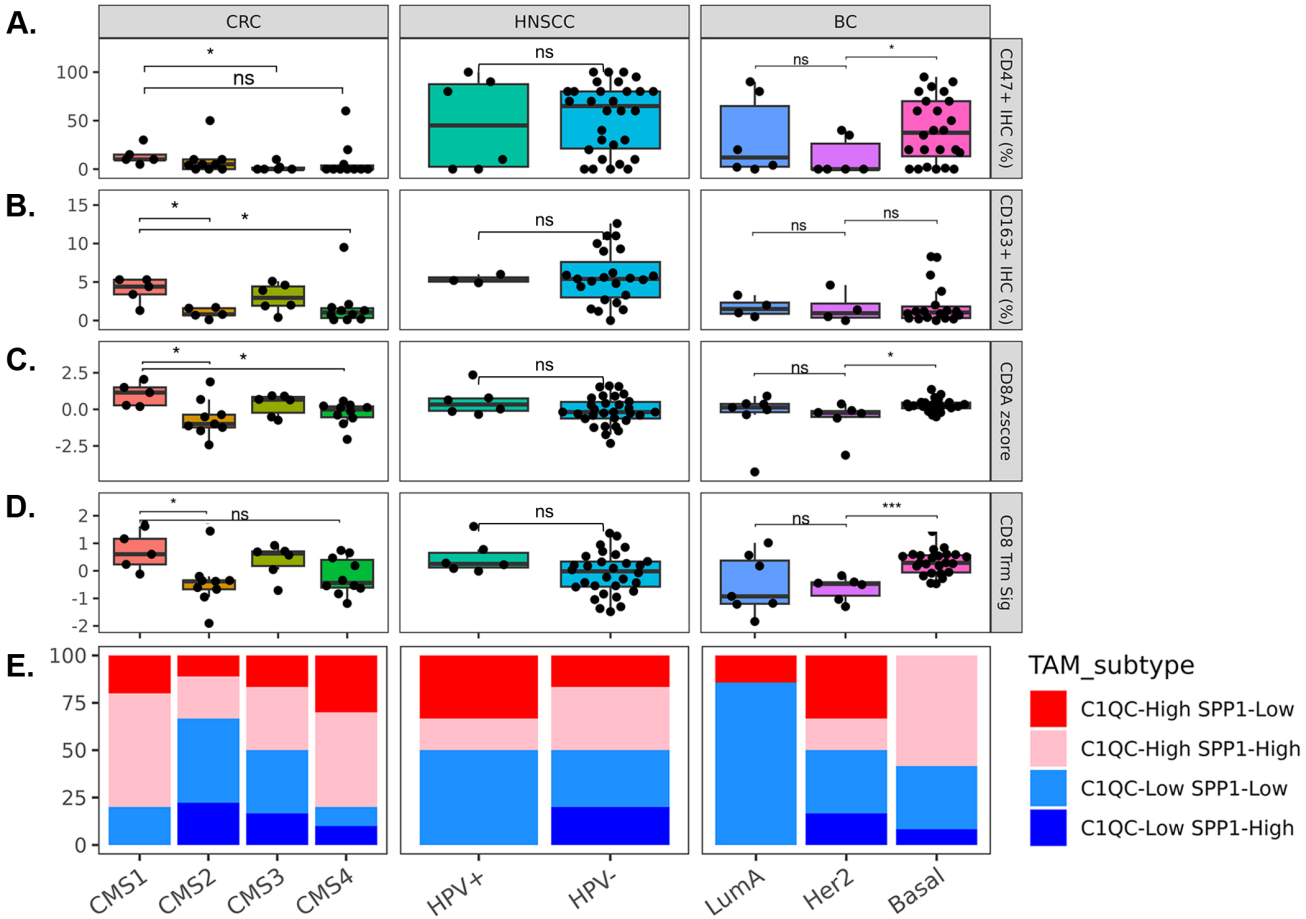
Tumor molecular subtypes influence TAM biology and CD47 expression. **(A)** CD47+ protein expression as percentage of total neoplastic cells plotted for each molecular subgroup. **(B)** CD163+ TAMs quantified using mIF plotted for each molecular subgroup. **C/D.** CD8A gene expression and CD8 Trm gene signature scores plotted for each molecular subgroup. **(E)** C1QC and SPP1 high and low groups were binned within each indication and the prevalence of TAM bins was plotted for each molecular subgroup. Statistical analyses performed using t-test. P values: * p < 0.05, ** p < 0.01, *** p < 0.001 and **** p < 0.0001.

CD47 protein expression, calculated as the percentage of tumor cells expressing CD47, has modest difference across the molecular subgroups (**Figure 5A**); the CMS1 CRC subtype demonstrated a statistically significant but subtle increase in CD47 expression compared to CMS3. In addition, the Basal-like subtype of BC was significantly higher CD47 expression compared to the Her2 positive subgroup. CD163^+^ macrophages (mIF panel) were the highest in the CMS1 molecular subgroup amongst the CMS subtypes (**Figure 5B**). CD8 T cells were quantified using CD8A gene expression and a Trm gene signature (**Figure 5C, D**) (CD8A, ITGAE (CD103), ZNF683 (HOBIT)); CMS1 contained the highest T cell abundance amongst the CMS subgroups, while the basal-like subgroup within BC had the highest T cell abundance. Finally, we calculated the prevalence for SPP1 and C1QC high versus low TAM bins within each cancer indication (**Figure 5E**). Notably, amongst molecular subgroups with lower levels of immune infiltration, CRC CMS2 showed lower proportion of C1QC TAMs and higher proportion of SPP1 TAMs, whereas BC Luminal A showed lower levels of both C1QC TAMs and SPP1 TAMs. The Basal-like BC subtype that is enriched for Triple Negative Breast cancers (TNBC) with the worst prognosis amongst BC subtypes is relatively balanced for C1QC TAM high and low samples. No significant differences were observed in HNSCC samples stratified by HPV status, although there was a limited number of HPV+ samples.

## Discussion

In the present study we developed immunohistochemistry tools for characterization of CD47 and macrophages in solid tumor samples. The data generated were integrated with TAM gene signatures and informative transcriptional markers and signatures, to gain an overall insight of CD47 and TAM biology across HNSCC, BC and CRC indications.

CD47 expression and tumor macrophage density were both highest in HNSCC compared to BC and CRC indications. In the context of PD-L1 checkpoint inhibition, it has been observed that PD-L1 expression and the presence of PD-1^+^ T cells in baseline tumor samples are both associated with objective response. In a similar fashion, it is possible that CD47 expression and tumor macrophage density may be predictive of clinical response to CD47-blockade. If so, it is notable that our current analysis highlights that HNSCC has the highest levels of these potential biomarkers. We observed significantly higher CD47 expression in CRC liver metastasis compared to primary tumors. This will be important context to capture clinically when evaluating the relationship between CD47 expression and response in trials exploring CD47-blockade. For example, when evaluating CD47 as a potential predictive biomarker, tumor samples taken from primary tumor and liver metastases should be analyzed separately, given the significant difference of CD47 expression in these two tissue sites. It will also be important to understand if patients with liver metastasis derive greater benefit from CD47-blockade.

Patients with high CD47 expression have been shown to demonstrate poorer outcomes compared to those with low CD47 expression in several indications, including breast cancer (7, 41), HNSCC (9) and acute myeloid leukemia (42). However, these data exploring CD47 as a negative prognostic biomarker have been generated using a variety of assay types and have been limited by small sample sizes. We used the Tempus real world database to evaluate CD47 expression as a prognostic biomarker in HNSCC, TNBC and CRC. The benefit of Tempus is the ability to control for lines of therapy and still achieve relatively high sample size. Here we selected only patient biopsies prior to front line therapy to achieve the largest possible sample set without confounding the analysis with multiple lines of therapy. Contrary to some other datasets, we did not observe any differences in survival between patients with high versus low CD47 RNA expression in any of the indications tested. However, further exploration of CD47 as a prognostic biomarker is warranted, especially if attempting to determine if CD47 expression is predictive of CD47-blockade activity in specific disease indications or in specific treatment settings.

The definition of M1 and M2 macrophages originally came from *in vitro* cultured monocyte-derived macrophages from peripheral blood. Single cell RNA-Seq datasets from tumor samples have uncovered significant differences between macrophages found *in vivo* and the M1/M2 phenotypes. In tumors, macrophages have been shown to originate either embryonically to establish tissue-resident macrophages, or through the infiltration of monocytes into tumor tissue which differentiate into macrophages within the tumor (43, 44). Tissue-resident macrophages replicate within tissue and tend to be pro-phagocytic. Beyond their origin, scRNA-Seq has defined several TAM subtypes including C1QC and SPP1 (11). We focused on C1QC and SPP1 TAM subsets as they appear to have clearly defined functional roles and demonstrate prognostic importance in a variety of settings (11, 45). C1QC TAMs are pro-phagocytic and may support anti-tumor immunity through cross presentation to CD4 T cells in the tumor (46). In addition, C1QC TAMs express a variety of tissue resident markers, indicating that they may have embryonic origin (46); C1QC macrophages can also be found in non-tumor tissues which is supportive of tissue residency. SPP1 TAMs are pro-angiogenic, and their presence in tumors correlates with poorer survival, due in part to increased tumor vasculature and reduced immune infiltration. In CRC, the ratio of SPP1 to C1QC TAMs is prognostic as patients with high-C1QC and low-SPP1 gene signature scores has the best survival. It will be fascinating to determine if SPP1 or C1QC gene signature scores are predictive of CD47-blockade activity or if either TAM subtype is predominantly modified by anti-CD47 treatment.

We demonstrated a correlation between CD47 expression and the C1QC TAM gene signature that was statistically significant in HNSCC and CRC and trended towards significant in BC. It has previously been shown that macrophage secreted IL-18 can lead to upregulation of CD47 (47). There is a similar link between PD-1^+^ T cells infiltrating tumors which leads to upregulation of tumor PD-L1 through the release of IFNγ (48). It will be intriguing to quantify CD47 expression following anti-CD47 blockade as this will hypothetically result in decreased CD47 expression as CD47^+^ tumor cells are phagocytosed. However, if macrophage activation leads to CD47 upregulation, we may observe an increase in CD47 following treatment, just as upregulation of PD-L1 is observed following checkpoint inhibition (49, 50).

Consensus molecular subgroups defined using genomics datasets have been developed across several solid tumor indications (36, 39). We utilized subgrouping algorithms in CRC and BC to categorize our samples and examined key macrophage signatures and other TME components across the various groups. In CRC, the most notable observation was that CMS1 and CMS3, which contain a high number of samples with microsatellite instability, have higher numbers of CD163+ macrophages and higher infiltrating T cells. These CMS subgroups typically respond better to immunotherapy. In BC, the basal-like subgroup appears to be the most immune infiltrated and has a higher prevalence for C1QC-high TAMs and the highest CD47 expression. Altogether, our data are consistent with the known characteristics of each of the previously characterized molecular subgroups, but there is limited differentiation of these groups when quantifying CD47 expression.

In conclusion, a CD47 IHC assay was developed and CD47 expression and prevalence patterns were quantified in HNSCC, BC and CRC tumor samples. We determined that HNSCC was the indication with the highest expression and prevalence of CD47, as well as highest density of TAMs, in primary tumor samples. Notably, CRC liver metastasis was observed to have significantly higher CD47 expression than primary CRC samples independent of treatment status. CD47 expression and TAM density/phenotype in baseline biopsies are both candidate biomarkers for CD47-blockade therapy. These assays will serve important roles when evaluating CD47-blockade clinical responses.

## Data Availability

The datasets presented in this study can be found in online repositories. The names of the repository/repositories and accession number(s) can be found in the Materials and Methods section.

## Supplementary material

Supplementary Figure 1 Correlation between digital image analysis and pathologist scoring for CD47 IHC**. (A).** Plot demonstrating the correlation between digital H-scores and pathologist H-scores across primary tumor samples from HNSCC, TNBC and CRC. **B.** Plot demonstrating the correlation between digital percent of cells positive for CD47 staining and pathologists’ assessment for percent positive.

Supplementary Figure 2 Development of a SIRPα chromogenic IHC assay. Solid tumor samples from HNSCC (n=44), BC (n=48) and CRC (n=48) were stained with an anti-SIRPα antibody. Images were scanned and analyzed using a digital pathology algorithm to calculate H-score. SIRPα expression was found on tumor cells as well as on immune cells. Tumor regions are denoted with dashed outlines and labeled T and stromal regions labeled S. Yellow triangles denote lymphocytes with strong membranous staining and black arrows denote macrophages.

Supplementary Figure 3 Evaluation of CD47 gene expression as a prognostic biomarker using Tempus real world evidence dataset. A. CD47 gene expression was stratified at the median within each indication, HNSCC, TNBC and CRC. Real world PFS was plotted for CD47-high and CD47-low populations. B. CRC samples were binned based on tumor sample location. Primary CRC tumors were compared separately from CRC liver metastases due to large observed expression differences observed by IHC. CD47 gene expression was stratified at the median for each CRC population and real world PFS was plotted. Shaded areas denote the 95% confidence intervals. Statistical comparisons by Mann-Whitney U test; p-values: NS p>=0.05, *p < 0.05, **p < 0.005, ***p < 0.0005.

Supplementary Figure 4 Clustering of tumor samples by molecular subgroup designations. **(A).** Samples within each indication were clustered using known molecular subtypes for CRC and BC or HPV status for HNSCC samples. Key gene signatures and individual genes were scored for each sample to visual the differences between clusters. **(B).** The CISH HPV RNAscope HR8 assay was used to determine HPV status; however, 3 samples were removed from analysis due to technical reasons. To provide a secondary HPV call for these samples, a published HPV+ transcript signature was used to predict the HPV status from the HNSCC RNA transcriptome data^43^.

Supplementary Figure 5 TAM signature development and application in translational dataset **(A).** Example of published scRNA-Seq data (32) showing expression of some published SPP1 TAM markers that are not specific to macrophages, but also highly expressed in cancer cells in UMAP feature maps. The purple color represents normalized signals and grey represents lack of signal. The TAM SPP1 score feature UMAP shows higher signal in the macrophage cluster located on the bottom left, where the SPP1 TAM cells are located. Feature UMAPs of SPP1 TAM markers show significant expression in cancer cell clusters, which are circled in red on each plot. A labeled UMAP plot is shown to annotate cell type identities (right) (**B).** C1QC and SPP1 TAM gene signatures from Zhang et al. (11) and a new SPP1 TAM signature from this study were tested using pseudo-bulk RNA-Seq samples generated from an independent scRNA-Seq dataset to evaluate the specificity of the signatures in a bulk RNA-Seq setting. Each column represents a pseudo-bulk sample that contains a known amount of TAM cells (the percent of TAM cells is labelled with a white box on the bars). The dot plots show the TAM signature score, which is calculated as the mean z-score of markers in the signature. Markers in the signatures are shown below the plot. The Zhang et al. SPP1 TAM signature and new SPP1 TAM signatures performed very similarly on the test data. Both signatures are most sensitive to presence of SPP1 TAM cells, although they also detect other TAM cells modestly. The C1QC TAM signature is very sensitive and specific to C1QC TAM cells. It does not detect other TAM subtypes significantly. **(C).** Expression of CD8A mRNA from CRC, BC & HNSCC (normalized by z-score within each cancer cohort) showing elevated CD8A in C1QC-High/SPP1-Low samples compared to C1QC-Low/SPP1-High samples.
