# Supplementary figures and images for: CD47 expression in solid tumors correlates with phagocytic tumor-associated macrophage gene signature
Source: Front Immunol. 2025 Dec 3;16:1699237. doi: 10.3389/fimmu.2025.1699237 (PMC12708551; doi:10.3389/fimmu.2025.1699237)

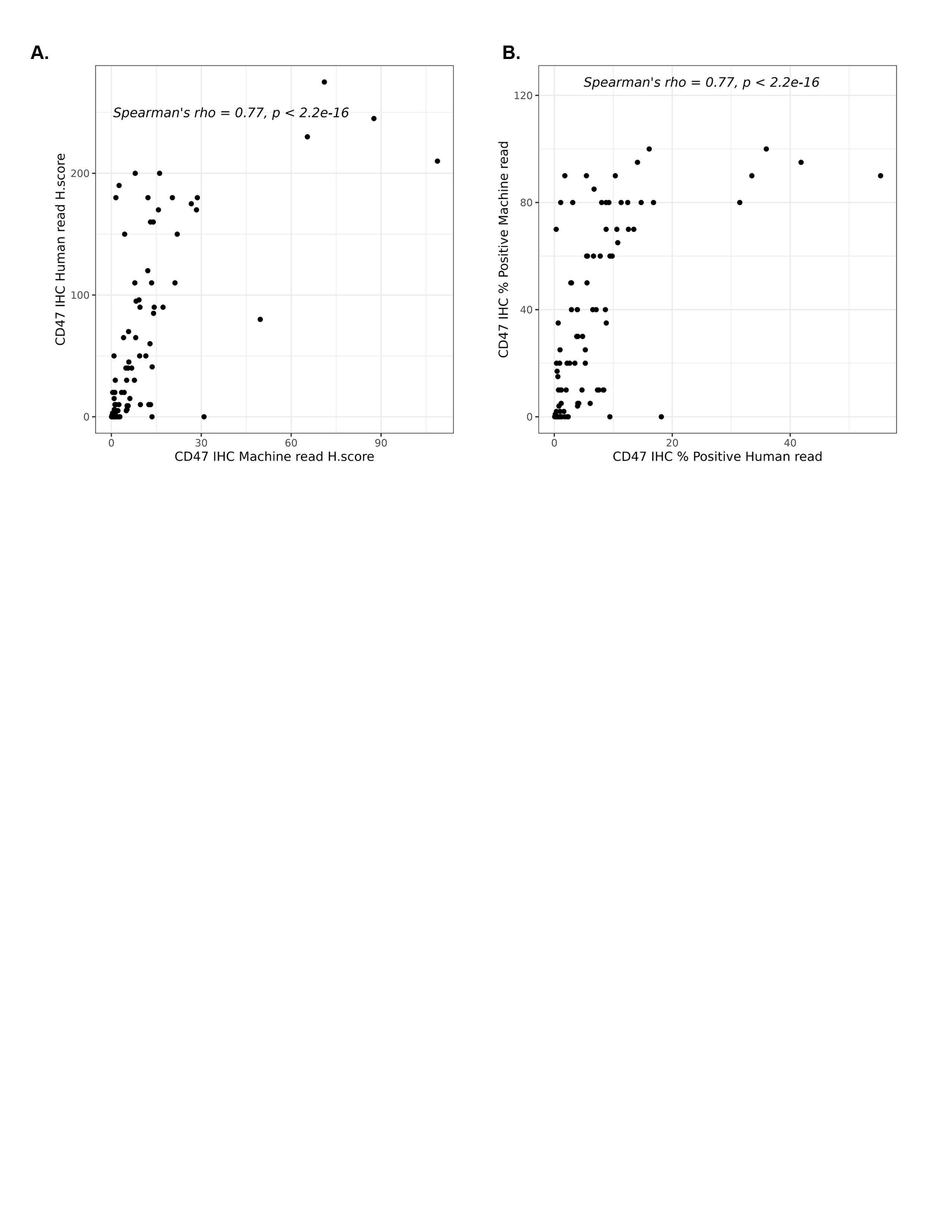

Supplement: Supplementary Figure 1 — Correlation between digital image analysis and pathologist scoring for CD47 IHC. (A). Plot demonstrating the correlation between digital H-scores and pathologist H-scores across primary tumor samples from HNSCC, TNBC and CRC. B. Plot demonstrating the correlation between digital percent of cells positive for CD47 staining and pathologists’ assessment for percent positive. [file Image1.jpeg]

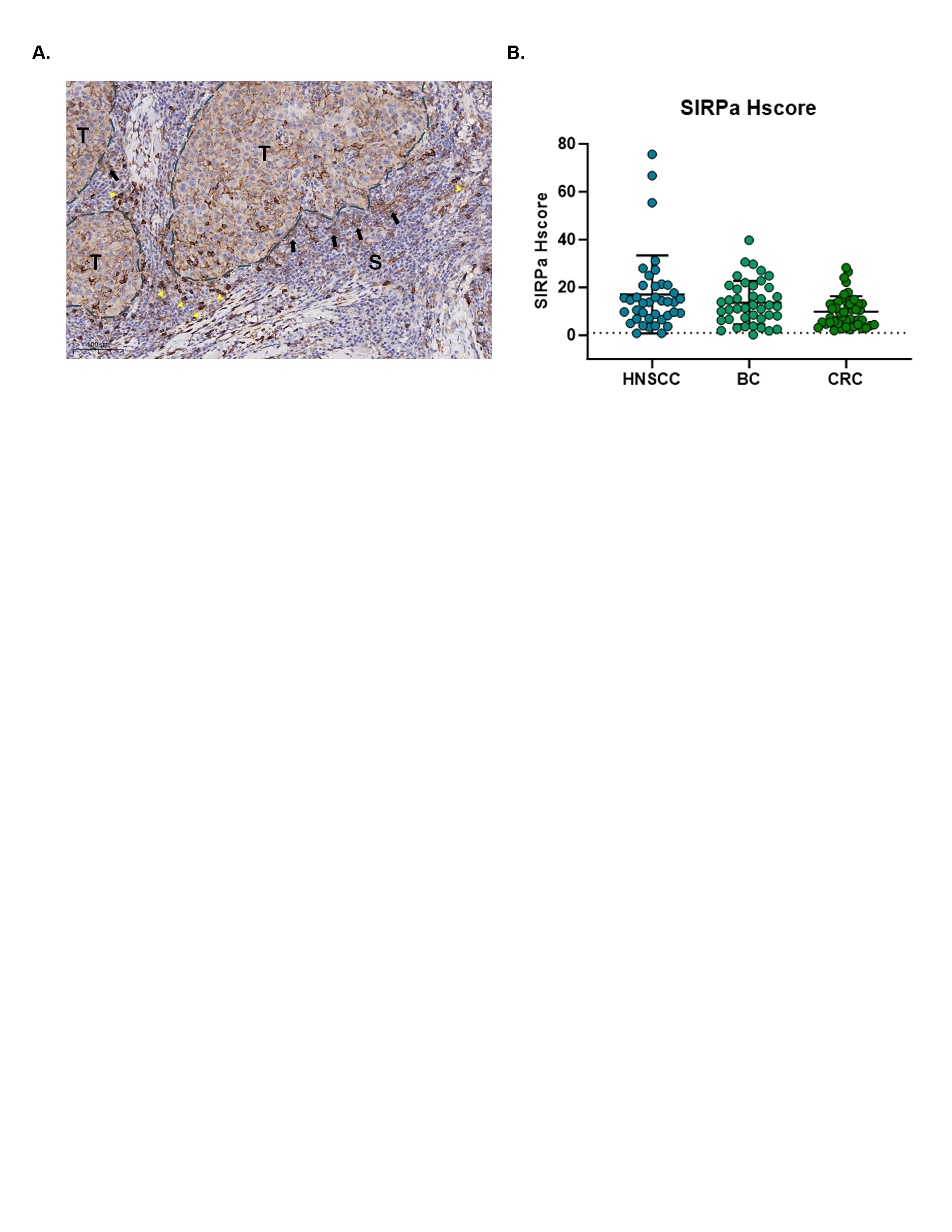

Supplement: Supplementary Figure 2 — Development of a SIRPα chromogenic IHC assay. Solid tumor samples from HNSCC (n=44), BC (n=48) and CRC (n=48) were stained with an anti-SIRPα antibody. Images were scanned and analyzed using a digital pathology algorithm to calculate H-score. SIRPα expression was found on tumor cells as well as on immune cells. Tumor regions are denoted with dashed outlines and labeled T and stromal regions labeled S. Yellow triangles denote lymphocytes with strong membranous staining and black arrows denote macrophages. [file Image2.jpeg]

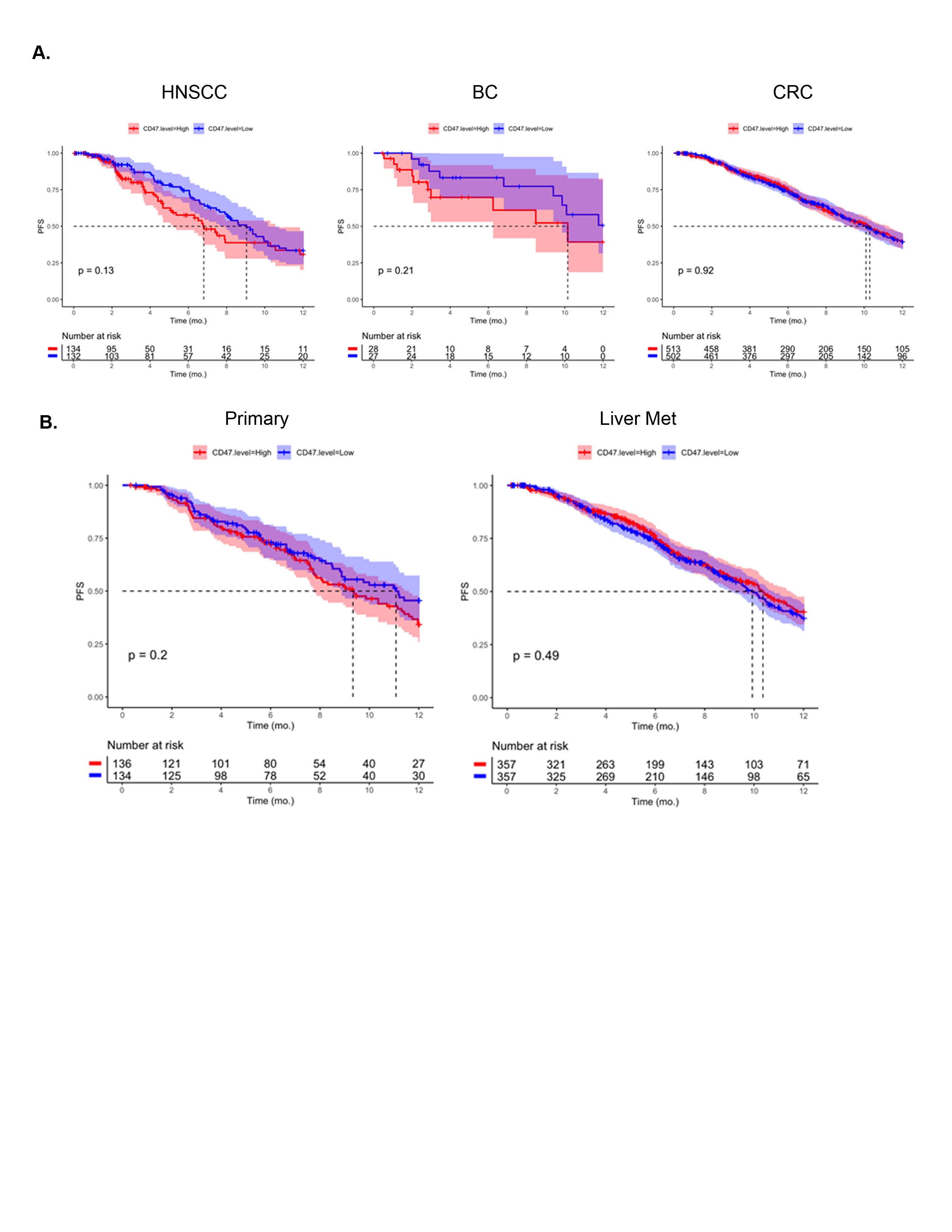

Supplement: Supplementary Figure 3 — Evaluation of CD47 gene expression as a prognostic biomarker using Tempus real world evidence dataset. A. CD47 gene expression was stratified at the median within each indication, HNSCC, TNBC and CRC. Real world PFS was plotted for CD47-high and CD47-low populations. B. CRC samples were binned based on tumor sample location. Primary CRC tumors were compared separately from CRC liver metastases due to large observed expression differences observed by IHC. CD47 gene expression was stratified at the median for each CRC population and real world PFS was plotted. Shaded areas denote the 95% confidence intervals. Statistical comparisons by Mann-Whitney U test; p-values: NS p>=0.05, *p < 0.05, **p < 0.005, ***p < 0.0005. [file Image3.jpeg]

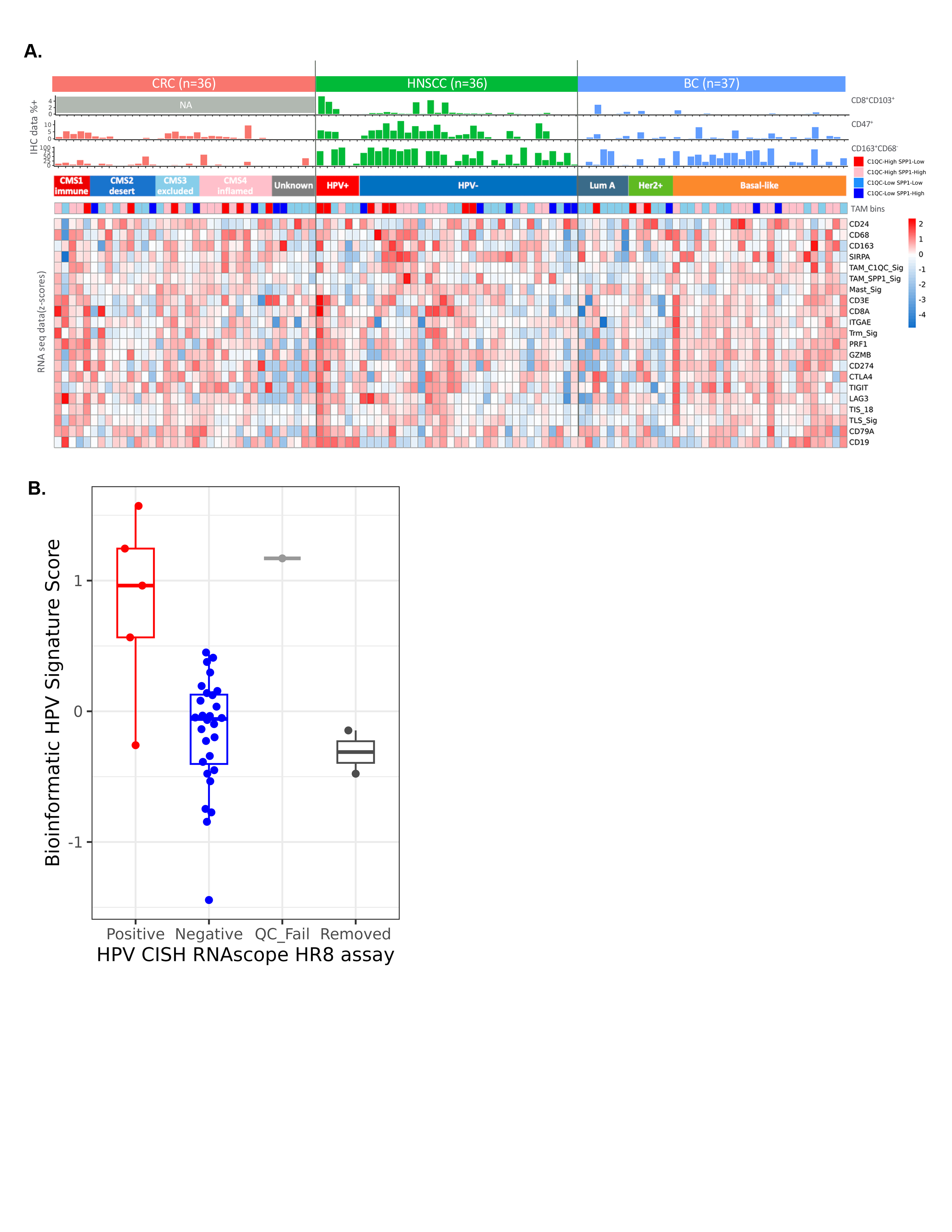

Supplement: Supplementary Figure 4 — Clustering of tumor samples by molecular subgroup designations. (A). Samples within each indication were clustered using known molecular subtypes for CRC and BC or HPV status for HNSCC samples. Key gene signatures and individual genes were scored for each sample to visual the differences between clusters. (B). The CISH HPV RNAscope HR8 assay was used to determine HPV status; however, 3 samples were removed from analysis due to technical reasons. To provide a secondary HPV call for these samples, a published HPV+ transcript signature was used to predict the HPV status from the HNSCC RNA transcriptome data43. [file Image4.jpeg]

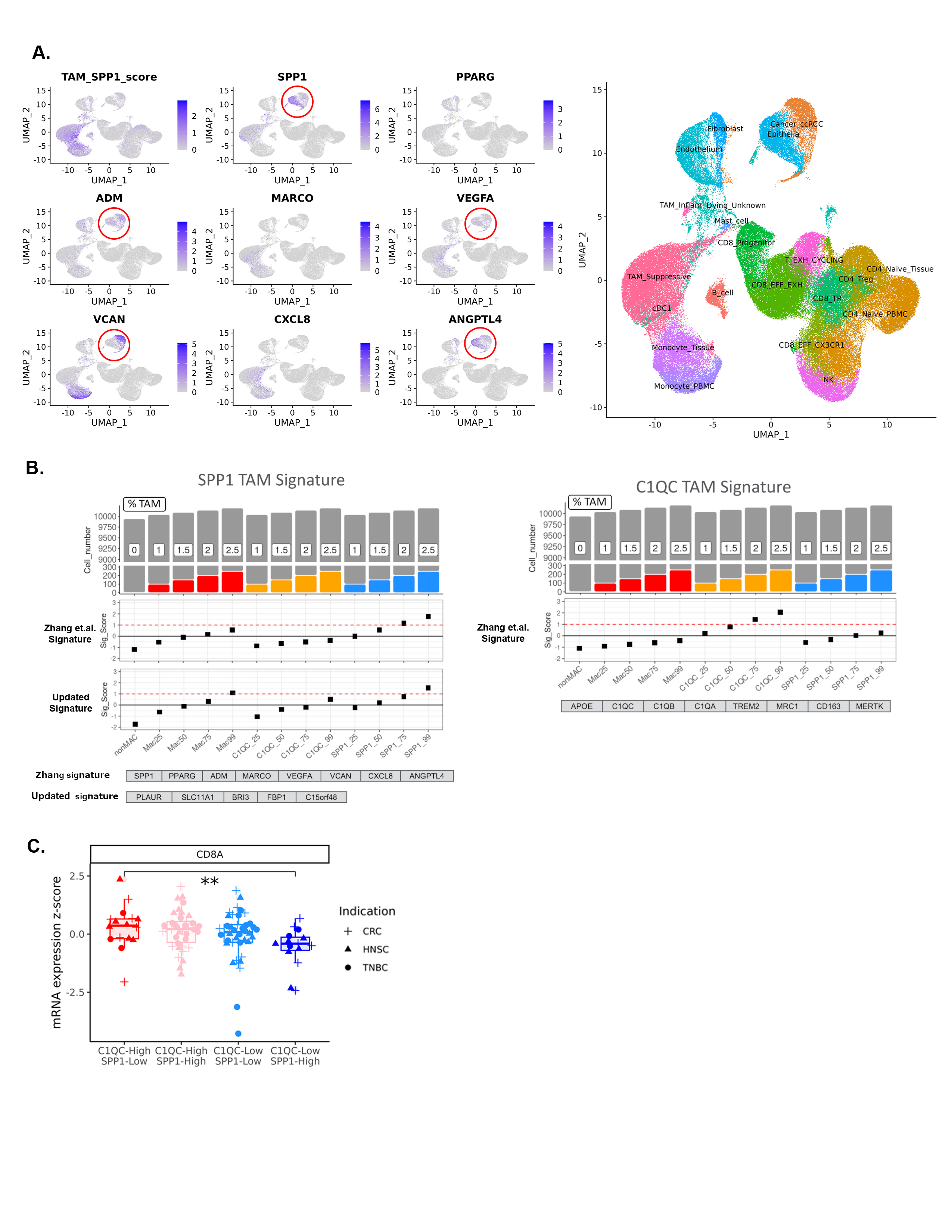

Supplement: Supplementary Figure 5 — TAM signature development and application in translational dataset (A). Example of published scRNA-Seq data (32) showing expression of some published SPP1 TAM markers that are not specific to macrophages, but also highly expressed in cancer cells in UMAP feature maps. The purple color represents normalized signals and grey represents lack of signal. The TAM SPP1 score feature UMAP shows higher signal in the macrophage cluster located on the bottom left, where the SPP1 TAM cells are located. Feature UMAPs of SPP1 TAM markers show significant expression in cancer cell clusters, which are circled in red on each plot. A labeled UMAP plot is shown to annotate cell type identities (right) (B). C1QC and SPP1 TAM gene signatures from Zhang et al. (11) and a new SPP1 TAM signature from this study were tested using pseudo-bulk RNA-Seq samples generated from an independent scRNA-Seq dataset to evaluate the specificity of the signatures in a bulk RNA-Seq setting. Each column represents a pseudo-bulk sample that contains a known amount of TAM cells (the percent of TAM cells is labelled with a white box on the bars). The dot plots show the TAM signature score, which is calculated as the mean z-score of markers in the signature. Markers in the signatures are shown below the plot. The Zhang et al. SPP1 TAM signature and new SPP1 TAM signatures performed very similarly on the test data. Both signatures are most sensitive to presence of SPP1 TAM cells, although they also detect other TAM cells modestly. The C1QC TAM signature is very sensitive and specific to C1QC TAM cells. It does not detect other TAM subtypes significantly. (C). Expression of CD8A mRNA from CRC, BC & HNSCC (normalized by z-score within each cancer cohort) showing elevated CD8A in C1QC-High/SPP1-Low samples compared to C1QC-Low/SPP1-High samples. [file Image5.jpeg]
